# The long noncoding RNA *lncR492* inhibits neural differentiation of murine embryonic stem cells

**DOI:** 10.1371/journal.pone.0191682

**Published:** 2018-01-24

**Authors:** Maria Winzi, Nuria Casas Vila, Maciej Paszkowski-Rogacz, Li Ding, Svenja Noack, Mirko Theis, Falk Butter, Frank Buchholz

**Affiliations:** 1 Medical Systems Biology, Faculty of Medicine Carl Gustav Carus, University Cancer Center, TU Dresden, Dresden, Germany; 2 Quantitative Proteomics, Institute of Molecular Biology (IMB) gGmbH, Mainz, Germany; 3 Max Planck Institute of Molecular Cell Biology and Genetics, Dresden, Germany; 4 German Cancer Research Center (DKFZ), Heidelberg and German Cancer Consortium (DKTK) partner site Dresden, Dresden, Germany; 5 National Center for Tumor Diseases (NCT), University Hospital Carl Gustav Carus, TU Dresden, Dresden, Germany; Centre for Stem Cell Research, UNITED KINGDOM

## Abstract

RNA interference (RNAi) screens have been shown to be valuable to study embryonic stem cell (ESC) self-renewal and they have been successfully applied to identify coding as well as noncoding genes required for maintaining pluripotency. Here, we used an RNAi library targeting >640 long noncoding RNAs (lncRNA) to probe for their role in early cell differentiation. Utilizing a Sox1-GFP ESC reporter cell line, we identified the lncRNA *lncR492* as lineage-specific inhibitor of neuroectodermal differentiation. Molecular characterization showed that *lncR492* interacts with the mRNA binding protein HuR and facilitates its inhibitory function by activation of Wnt signaling. Thus, lncRNAs modulate the fate decision of pluripotent stem cells.

## Introduction

Embryonic stem cells (ESC) are characterized by their ability of long-term self-renewal as well as their potential to differentiate into each cell type of the embryo proper. After the first isolation of embryonic stem cells from the mouse blastocyst [1, 2] the research community has achieved a reasonable understanding of the regulatory mechanisms controlling self-renewal of ESC [3]. However, knowledge about the transition from pluripotency to the first lineage commitment is still less well understood.

Recent sequencing approaches have shown that the majority of the genome is transcribed [4]. Among the identified transcripts are RNAs that are transcribed by Polymerase II, usually 5’ capped, polyadenylated and spliced but have little or no protein coding potential [5, 6]. With a transcript length of >200 nucleotides they are defined as long noncoding RNAs (lncRNA). LncRNAs can originate intergenically or are transcribed from a promoter shared with the protein-coding gene. Recent research revealed very diverse mechanisms how lncRNA function: e.g. by chromatin remodeling, histone modification, DNA methylation or interaction with transcription factors but also as scaffolds for protein assembly, as miRNA sponges, or posttranscriptional gene regulators by controlling alternative splicing or influencing degradation [7].

Large-scale functional studies have identified numerous lncRNAs that play a regulatory role in the maintenance of pluripotency [8–10]. It has been shown that lncRNAs are under tight control of important pluripotency-associated transcription factors, but also feed back into the circuit of self-renewal and differentiation [8, 11]. For instance, the lncRNA *TUNA* was identified as sustainer of pluripotency but was also required for neural differentiation [10]–highlighting the extensive and context-dependent role of lncRNAs.

To identify specific neural differentiation regulating lncRNAs, we performed a large-scale loss-of-function RNAi screen in a Sox1-GFP ESC reporter line. We discovered the lncRNA *lncR492* as an inhibitor of neural differentiation, which exerts its function by interacting with the mRNA binding protein HuR and activating Wnt signaling, thereby blocking ectodermal differentiation in a lineage specific manner.

## Materials and methods

### Cell culture and high-throughput screen

Austin Smith and Konstantinos Anastassiadis kindly provided the Sox1-GFP and Oct4-GFP cell lines. The Foxa2-GFP and T-GFP cell lines were generated in the BAC TransgeneOmics project [12] and kindly provided by Ina Poser. R1/E ESCs were obtained from the transgenic core facility of the Max Planck Institute of Molecular Cell Biology and Genetics.

Sox1-GFP, Foxa2-GFP, T-GFP ESC and R1/E ESC lines were cultured on gelatin-coated plates in DMEM (high glucose, GlutaMAX^™^) supplemented with 1x N2, 1x B27, 1x NEAA, 1x Penicillin/Streptomycin, 50 μM 2-mercaptoethanol (all from ThermoFisher Scientific), 3 μM CHIR99021, 1 μM PD0325901 (both from Calbiochem) and LIF (generated in house). For differentiation towards the ectoderm cells were cultured in N2B27 containing medium without the two inhibitors and LIF. For differentiation into endoderm (Foxa2-GFP ESC) and mesoderm (T-GFP ESC), the two inhibitors and LIF were replaced by 30 μg/ml ActivinA (MPI protein facility) or 10 μg/ml BMP4 (R&D Systems), respectively. The cells were seeded on gelatin-coated dishes with a density of 15000 cells/cm^2^ and grown for 4 to 5 days before they were harvested for experiments.

Oct4-GFP ESC were additionally cultured on gelatin-coated plates in DMEM (high glucose, GlutaMAX^™^) supplemented with 10% Pansera ES FBS (PAN Biotech), 1x NEAA, 1x Penicillin/Streptomycin, 50μM 2-mercaptoethanol (all from ThermoFisher Scientific) and LIF (MPI-CBG protein facility).

For SILAC-labeling the standard DMEM was replaced with SILAC-DMEM medium (-Arg, -Lys) supplemented with 40 mg/mL ^13^C_6_^15^N_4_ l-arginine and 80 mg/mL ^13^C_6_^15^N_2_ l-lysine (Sigma Isotec or Euris-top) or the corresponding non-labeled amino acids, respectively. All other supplements were maintained as described. Cells were grown for five passages, lysed and checked for successful label incorporation by MS.

EsiRNAs were synthesized as described previously [13]. For the lncRNA screen cells were transfected with esiRNAs using Lipofectamine 2000 (ThermoFisher Scientific) as previously described [14]. GFP fluorescence and cell numbers were measured after 96 hours of differentiation using a FACS Calibur (BD biosciences) equipped with an HTS loader for high throughput analysis.

### Transient overexpression

The cDNAs for lncR492 and HuR were amplified by PCR introducing *NheI* and *XhoI* restriction sites in the flanking regions. After sequencing, the PCR products were cloned into the pCGIT destination vector under the CAG promoter (a kind gift from Prof. Palle Serup). pCGIT without insert was used as control (EV—empty vector).

ESCs were transiently transfected with the overexpression or control plasmids using Lipofectamine 2000 (ThermoFisher Scientific). 50 ng of plasmid DNA were combined with 0.2 μl Lipfectamine 2000 and incubated in 50 μl OptiMEM for 10 minutes. The transfection mix was added to one well of a 96-well plate. 4500 cells per well in 200 μl N2B27 medium supplemented with 2i+LIF were seeded on top. After 24h differentiation was initiated as described above.

### RT-PCR

Total RNA was isolated by using the RNeasy Mini kit (QIAGEN). For each RT reaction 1 μg of RNA was reverse transcribed with SuperScript III Reverse Transcriptase (ThermoFisher Scientific) utilizing oligo(dT)_18_ primer. Standard RT-PCR reactions were performed with a MyTaq^™^ Red DNA Polymerase (Bioline). Quantitative RT-RCRs (qRT-PCR) were run with the SYBR Green qPCR kit (Abgene) on an CFX96 Touch^™^ Real-Time PCR Detection System (Bio-Rad). Measured transcript levels were normalized to *Gapdh*. Samples were run in duplicates. Primers used are listed below.

For endonuclease treatment 5 μg RNA were treated with a 5’-phosphate-dependent exonuclease according to manufactor’s instructions (mRNA-ONLY^™^ Eukaryotic mRNA Isolation Kit, EpiCentre). Subsequent 500 ng of RNA were reverse transcribed as described above.

The forward and reverse primer sequences (written 5’ to 3’) were as follows:

*Gapdh*, ACTCCACTCACGGCAAATTC, GGATGCAGGGATGATGTTCT
*lncR492*, GCTGCTGTTTCACACCCAAG, TGACTAGGCGATCCTGACCA
*Sox1*, CCTTGCTAGAAGTTGCGGTC, TCACTCAGGGCTGAACTGTG
*Srrm4*, TCTCGTCGAAGTCCCAGCTA, CACTGGTTATCCTCCGAGCC
*Oct4*, AGAGGGAACCTCCTCTGAGC, TGATTGGCGATGTGAGTGAT
*Brachyury (T)*, GAACAGCTCTCCAACCTATG, AGACTGGGATACTGGCTAGAG
*Foxa2*, GCTGCAGACACTTCCTACTAC, GGACACAGACAGGTGAGACT
*Pax6*, CACCAGACTCACCTGACACC, TCACTCCGCTGTGACTGTTC
*Nestin*, GCAGGAGAAGCAGGGTCT, AGGTGCTGGTCCTCTGGT
*Nanog*, GGAAGCAGAAGATGCGGACT, ATGCGTTCACCAGATAGCCC
*Malat1*, GTTTGTGATTGGAGCCGAG, AAGGGAGGGGAGAGAGAACA
*U1 snRNA*, GCGCGATTTGGCAAGATGA, TTCTTCCCGGGTTTCTGCTC

### Northern blot

The sequences of the gene-specific Northern blot probes are listed below (written 5’ to 3’). Two probes per gene were mixed together. Northern blot was performed according to the DIG Northern Starter Kit’s (Roche) manual. Briefly, RNA was separated on a pre-cast 1% agarose gel at 35V for 3.5h in 1x MOPS buffer. The separated RNA was blotted on a nitrocellulose membrane (GE Healthcare) and cross-linked by UV-light. The membrane was prehybridized with DIG easy Hyb for 30 minutes at 68°C, followed by an hybridization step with a DIG-labeled, gene-specific RNA probe mix diluted in DIG easy Hyb over night at 68°C. Unspecific binding was eliminated by several stringency washes. Thereafter the membrane was blocked, incubated with a DIG-specific antibody and washed. Finally, the membrane was incubated with CDP-*Star* solution, exposed to Amersham Hyperfilm (GE Healthcare), which was developed using an OPTIMAX (PROTEC).

*lncR492* probe 1

UCAGAACCCAGCACACUGUCAGCCUCAGAGCAUACAAUCUUUUGAGUGAGUGAGCUAGAUUUGACAACGAUGCUUGAUGCUUGCGAGCAUAUGAGGGGCUCGCAGCCUCUUCCUAGGCACUACUGUCUCCCUCCGGAGACGCCUCCUGGCCUCCUUGAUUAUGAACACCUUUGCUAAUGCUCACAGUCUUCUUGUUCUCAUGGCCCUUGGAGGAUAUGUGCAGUGGUGAACACAGCU

*lncR492* probe 2

AUGGCUUUUUCCUCGCUACAGUUCAGAGUCGCCACAAUCACAGGGGGCCGGGGAAGAUCACCAGCAAUCAGUGUUCAAACGGCCCAAAGAGAUUGUUCUGAGUCUCCUUGCCACUCCGCUGAUGGAGUAGGGCCUUCUAAUUGGCCCUUCUGUCUCUCCUGCCUCGCCCUUCCAAUCUUUUGCCUUCUUGGCAGCUAGGUUCAUGCUUAUAGACCAUUUUUCGGUGAGUCAUUGCCUUGAUUUAAAACCCUUCGGCUUCCACUUGAGACUUGAGCCAUCCAGGCUCCCUGCCAUGGAGGGAGAGGGCAGUCAUGAUCUGGUUUUGGCCAUCCAUCUCUCUCCUCUAUCCUGUUCCCUGAGUCCUUGUUCUGAGCAUCUACCUGGGGCAUCCUUCCUUCCCACUAACUCCUGUAUCAUGUCCCUGGACCACUCCCCACCUGACUGGUCCUCUGUGUUUUCAGAGUAGCUGAUUUCUUUUGCACUGCCUAGCGCCACUGGGAACAGACAUCGUCUGUGAGCUUUGGGAAGG

*Gapdh* probe 1

CAGCGAACUUUAUUGAUGGUAUUCAAGAGAGUAGGGAGGGCUCCCUAGGCCCCUCCUGUUAUUAUGGGGGUCUGGGAUGGAAAUUGUGAGGGAGAUGCUCAGUGUUGGGGGCCGAGUUGGGAUAGGGCCUCUCUUGCUCAGUGUCCUUGCUGGGGUGGGUGGUCCAGGGUUUCUUACUCCUUGGAGGCCAUGUAGGCCAUGAGGUCCACCACCCUGUUGCUGUAGCCGUAUUCAUUGUCAUACCAGGAAAUGAGCUUGACAAAGUUGUCAUUGAGAGCAAUGCCAGCCCCGGCAUCGAAGGUGGAAGAGUGGGAGUUGCUGUUGAAGUCGCAGGAGACAACCUGGUCCUCAGUGUAGCCCAAGAUGCCCUUCAGUGGGCCCUCAGAUGCCUGCUUCACCACCUUCUUGA

*Gapdh* probe 2

AAGCAGUUGGUGGUGCAGGAUGCAUUGCUGACAAUCUUGAGUGAGUUGUCAUAUUUCUCGUGGUUCACACCCAUCACAAACAUGGGGGCAUCGGCAGAAGGGGCGGAGAUGAUGACCCUUUUGGCUCCACCCUUCAAGUGGGCCCCGGCCUUCUCCAUGGUGGUGAAGACACCAGUAGACUCCACGACAUACUCAGCACCGGCCUCACCCCAUUUGAUGUUAGUGGGGUCUCGCUCCUGGAAGAUGGUGAUGGGCUUCCCGUUGAUGACAAGCUUCCCAUUCUCGGCCUUGACUGUGCCGUUGAAUUUGCCGUGAGUGGAGUCAUACUGGAACAUGUAGACCAUGUAGUUGAGGUCAAUGAAGGGGUCGUUGAUGGCAACAAUCUCCACU

### Nuclear fractionation

Approx. 10x10^6^ cells were washed with ice-cold PBS. Cells were resuspended in ice-cold, protease inhibitor (Roche) containing HD-buffer (10 mM HEPES, 1 mM DL-Dithiotreirol) and incubated on ice for 10 minutes. IGEPAL was added to a final concentration of 0.5%. Quickly citric acid (100 mM) was added to a final concentration of 1 mM. The cells were vortexed vigorously. The nuclei were separated from the cytoplasmic fraction by gentle centrifugation. Total RNA was isolated with the PARIS Kit (Ambion). 1 μg of RNA was reverse transcribed with SuperScript III Reverse Transcriptase (ThermoFisher Scientific) utilizing oligo(dT)_18_ primer. qRT-RCR were run as described above.

### Fluorescent *in-situ* hybridization (FISH)

Sox1-GFP ESC were transfected with esiRNA using Lipofectamine2000 (Thermo Fisher Scientific) and seeded on gelatin-coated chambered slides (Ibidi). After 48 h RNA-FISH was performed as described earlier [9]. The sequence of the Northern blot *lncR492* probe 2 was used as FISH probe.

### Immunofluorescence

For immunofluorescent stainings cells were fixed with 4% paraformaldehyde for 10 minutes at room temperature. All following steps were performed at room temperature. After washing with PBS cells were blocked and premeabilized with 10% FCS in staining buffer (0.3%TritonX-100 in PBS) for 30 minutes. The primary antibody rabbit-anti-Tubb3 (Abcam, ab18207) was diluted 1:1000 in staining buffer and incubated for 1 h. After washing the cells were incubated with donkey-anti-rabbit-Cy3 secondary antibody (Jackson ImmunoResearch Laboratories) and DAPI (Sigma) for 30 minutes. Finally the cells were imaged using a Zeiss Axiovert 200M microscope equipped with an ApoTome (Zeiss). Images are projections of the maximum intensity. Total numbers of nuclei as well as Tubb3+ cells were counted manually.

### Mass spectrometry

#### RNA bait preparation and pull-down

To create the 450 bp RNA baits, forward primers containing the T7 promoter sequence and reverse primers with the S1 aptamer sequence were used in a PCR amplification reaction on the pCAGGS plasmid. A fragment of the human PTPN13 mRNA of equal length (450 bp) was used as a control bait. The primer sequences are written 5’ to 3’.

AK016992 (1–450 bp) + T7 forward primer, CGTTAATACGACTCACTATAGGCTCGAGATCCAGCTGGGCACAGGC.

AK016992 (1–450 bp) + BioApt reverse primer, CATGGCCCGGCCCGCGACTATCTTACGCACTTGCATGATTCTGGTCGGTCCCATGGATCCAACTCTGCTGATCGGATCTGTCCTC.

AK016992 (451–900 bp) + T7 forward primer, CGTTAATACGACTCACTATAGGGGCTCCCGGACCTTCCCAAAG.

AK016992 (451–900 bp) + BioApt reverse primer, CATGGCCCGGCCCGCGACTATCTTACGCACTTGCATGATTCTGGTCGGTCCCATGGATCCAGATTGTTCTGAGTCTCCTTGCCAC.

AK016992 (901–1350 bp) + T7 forward primer, CGTTAATACGACTCACTATAGGCTTTGGGCCGTTTGAACACTG.

AK016992 (901–1350 bp) + BioApt reverse primer, CATGGCCCGGCCCGCGACTATCTTACGCACTTGCATGATTCTGGTCGGTCCCATGGATCCTCTAGACGGGTACAATGCCTTC.

AK016992 (220–670 bp) + T7 forward primer, CGTTAATACGACTCACTATAGGAGGGCCATGAGAACAAGAAG.

AK016992 (220–670 bp) + BioApt reverse primer, CATGGCCCGGCCCGCGACTATCTTACGCACTTGCATGATTCTGGTCGGTCCCATGGATCCGTTTTGGCCATCCATCTCTCTC.

AK016992 (671–1120 bp) + T7 forward primer, CGTTAATACGACTCACTATAGGCAGATCATGACTGCCCTCTCC.

AK016992 (671–1120 bp) + BioApt reverse primer, CATGGCCCGGCCCGCGACTATCTTACGCACTTGCATGATTCTGGTCGGTCCCATGGATCCCGCTTGGGTGTGAAACAGCA.

PTPN13 + T7 forward primer, CGTTAATACGACTCACTATAGG CAATATATTTTCTGCTATCAAGTC.

PTPN13 + BioApt reverse primer, CATGGCCCGGCCCGCGACTATCTTACGCACTTGCATGATTCTGGTCGGTCCCATGGATCCCTTTATTAAAATATTGGAAAACATTTTTG.

The PCR products were used for *in vitro* transcription according to the manufacturer’s protocol (Fermentas). Successful transcription was monitored by agarose gel electrophoresis and RNA concentration was quantified by A280 absorbance on a Nanodrop system (Peqlab). 25 μg of each S1-tagged RNA was coupled to paramagnetic streptavidin C1 beads (Dynabeads MyOne, Invitrogen) in RNA binding buffer (100 mM NaCl, 10mM MgCl_2_, 50mM Hepes·HCl (pH 7.4), 0.5% IGEPAL CA-620) and incubated on a rotation wheel for 30 min at 4°C. RNA-bound beads were washed 3 times with RNA washing buffer (250 mM NaCl, 10mM MgCl_2_, 50mM Hepes·HCl (pH 7.4), 0.5% IGEPAL CA-620), prior to incubation with 400 μg nuclear extract, with 20 μg competitor yeast tRNA (Invitrogen) added, for 30 min at 4°C with gentle agitation. After mild washing, SILAC heavy and light fractions were combined 1:1 and samples were boiled in 1x LDS buffer (Invitrogen) and separated on a 4–12% NuPAGE Novex Bis-Tris precast gel (Life Technologies) at 180 V in 1x MOPS.

#### MS sample preparation

Coomassie stained gels were cut in one slice and destained with 50% EtOH/25 mM ammonium bicarbonate (ABC). The resulting gel pieces were dehydrated with 100% acetonitrile (ACN) and dried for 5 min in a concentrator (Eppendorf). Samples were incubated with reduction buffer (10 mM DTT/50 mM ABC) for 30 min at 56°C and further alkylated for 30 min in the dark with iodoacetamide (50 mM IAA/50 mM ABC). Gel pieces were completely dehydrated with ACN and covered in trypsin solution (1 μg trypsin per sample). Proteins were digested over night at 37°C and peptides were extracted twice by incubation with extraction buffer (3% TFA and 30% ACN) for 15 min. The gel pieces were dehydrated with 100% ACN and the extracted volume reduced to aproximately 150 μl in a concentrator (Eppendorf). Extracted peptides were desalted in StageTips (PMID:17703201) using two layers of C_18_material (Empore).

#### MS measurement and data analysis

Eluted peptides were injected via an autosampler into an uHPLC (EASY-nLC 1000, Thermo) and loaded on a 25 cm capillary (75 μm inner diameter; New Objective) packed in-house with Reprosil C18-AQ 1.9 μm resin (Dr. Maisch) for reverse-phase chromatography. The EASY-nLC 1000 HPLC system was directly mounted to a Q Exactive Plus mass spectrometer (Thermo). Peptides were eluted from the column with a 90 min optimized gradient from 2 to 40% ACN with 0.1% formic acid at a flow rate of 200 nL/min. Chromatography was stabilized with a column oven set-up operating at 40°C (Sonation). The heated capillary temperature was set to 250°C. Spray voltage ranged from 2.2–2.4 kV. The mass spectrometer was operated in data-dependent acquisition mode with one MS full scan and up to ten triggered MS/MS scans using HCD fragmentation (PMID:17721543). MS full scans were obtained in the orbitrap at 70,000 resolution with a maximal injection time of 20 ms, while MS/MS scan resolution was set to 17,500 resolution and maximal injection for 120 ms. Unassigned and charge state 1 were excluded from MS/MS selection and peptide match was preferred. Raw files were processed with MaxQuant (version 1.5.2.8.) (PMID:19029910) and searched against human UNIPROT annotated protein database provided with MaxQuant (81,194 entries) using the Andromeda search engine (PMID:21254760). Carbamidomethylation was set as a fixed modification, while acetyl (N-term protein) and oxidation (Met) were considered as variable modifications. Trypsin (specific) was selected as enzyme specificity with maximal two miscleavages for MaxQuant analysis. Proteins were quantified with at least 2 ratio counts based on unmodified unique and razor peptides. Known contaminants and reverse hits were removed before plotting the protein ratios of the forward and reverse experiments in R (version 3.2.2).

RNA pull-down experiments were performed 3 times—once for mass spectrometry and twice for western blot to confirm the enrichment of HuR.

### Luciferase assay

Cells were co-transfected with Super 8x TOPFlash or Super 8x FOPFlash luciferase plasmid together with either target-specific esiRNA or overexpression plasmid as well as pCMV renilla plasmid to correct for transfection efficiency. Cells were cultured in N2B27+2i+LIF. 48 h after transfection cells were lysed and bioluminescence was analysed using the Dual-Luciferase^®^ Reporter Assay (Promega) according to the manufacture’s instruction and the EnVision Multilabel Reader (PerkinElmer).

### Western blot

Total cell lysates were harvest 72h after transfection using RIPA lysis buffer. 20 μg of each protein sample were loaded and analysed by western blot using a mouse anti-HuR monoclonal antibody (Santa Cruz Biotechnology, sc-5261, 1:1000), rabbit anti-Tubb3 (Abcam, ab18207, 1:1000) and mouse anti-Gapdh (Novus Biologicals, NBP2-27103, 1:5000) as primary antibodies. As secondary antibodies HRP-conjugated species-specific antibodies (Biorad, 1:5000) were used. For quantitative analysis species-specific secondary antibodies from LI-COR Biosciences have been used. Blots were scanned and analysed with Odyssey.

### Statistical analysis

The results are shown as mean ± standard deviation (SD) as indicated in the figure legends. Statistical significance was calculated using the unpaired Student’s *t*-test.

## Results

### RNAi screen identifies lncRNA regulating ectodermal differentiation

**Fig 1:**
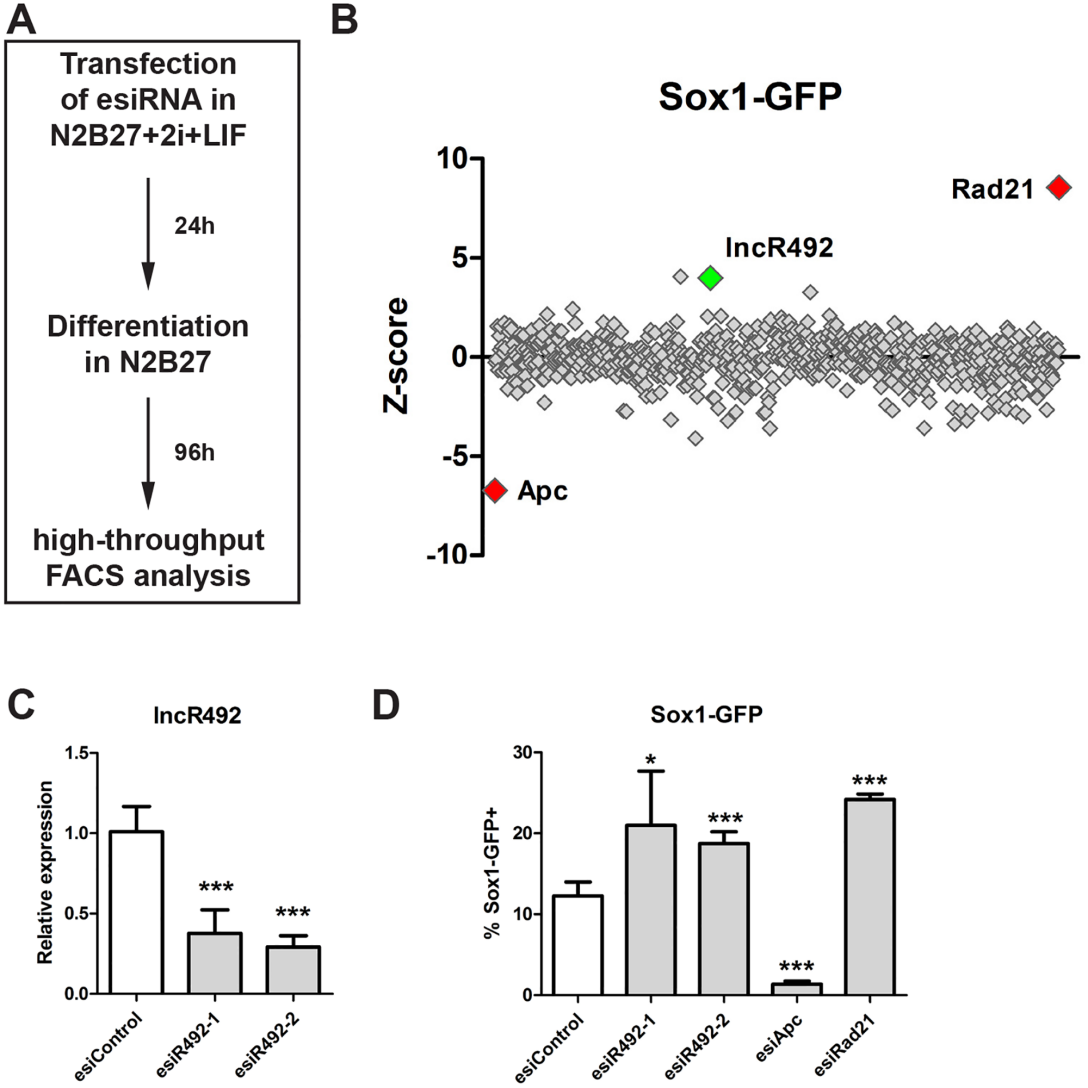
RNAi screen for Sox1-regulating lncRNAs. (A) Schematic overview over screening setup. Sox1-GFP cells were transfected with esiRNAs targeting lncRNAs under self-renewing conditions. The next day differentiation was initiated by media change and 4 days after differentiation Sox1-GFP expression was analysed by FACS. (B) Z-score values of the primary screen. Rad21 and Apc knock-down were used as controls. (C) Knock-down efficiency of *lncR492* targeting esiRNA was tested by qRT-PCR. Data presents the mean ± SD of three independent experiments. Data was normalized to esiControl. (D) FACS analysis of % Sox1-GFP positive cells after esiRNA transfection and 4 days of differentiation. Validation of screening results with two independent esiRNAs. Apc and Rad21 were targeted as controls. Data presents mean ± SD of 5 independent experiments. * p<0.05; ** p<0.01; *** p<0.001; n.s.–not significant.

Sox1 is one of the earliest transcription factors marking the neural ectoderm [15] and Sox1-GFP ESCs have been demonstrated to be capable of differentiating into neural progenitors when cultured in serum-free N2B27 supplemented medium without LIF (or other pluripotency maintaining cues) [16]. To identify lncRNAs that regulate ectodermal differentiation we used an established esiRNA library targeting 642 lncRNAs [9] in differentiating Sox1-GFP mouse ESC. Sox1-GFP ESCs were cultured in medium supplemented with N2B27+2i+LIF under self-renewing conditions. Cells were then transfected with esiRNAs in 384-well plates while still maintaining pluripotency growth conditions. At 24 hours post transfection the medium was changed to N2B27 medium to stimulate neural differentiation. To identify differences in the differentiation behavior, the number of Sox1-GFP+ cells was analysed by FACS in a high-throughput manner (Fig 1A). This approach allowed identifying both inducers (increased fraction of Sox1 positive cells) and inhibitors (reduced fraction of Sox1 positive cells) of ectodermal differentiation in a single screening setup. Knock-down of Apc, a component of the ß-catenin destruction complex, results in an activation of Wnt signalling, which has previously been shown to negatively affect neuroectodermal differentiation in ESC [17, 18] and was therefore used as one of the controls in our screen. As a second control Rad21 was used, as its knock-down leads to a general differentiation into all lineages, including Sox1-positive ectodermal cells. [14, 19, 20].

The primary screen resulted in 9 lncRNA hits with a Z-score > 3 or > -3 scoring in two biological replicates, whereof 3 up-regulated and 6 down-regulated the number of Sox1-GFP+ cells after knock-down (Fig 1B, S1 Table). For the validation screen a second independent, non-overlapping esiRNA for each hit was designed and tested. With stringent criteria, 3 of our 9 candidates scored in 6 out of 7 replicates demonstrating a robust phenotype. Among the validated screening hits *lncR492* (accession no. *AK016992*) had the strongest effect (Z-score (1^st^ esiRNA) = 4.0 and Z-score (2^nd^ esiRNA) = 5.4; Fig 1B). To verify the results obtained in the screen we tested the knock-down efficiency of the utilized esiRNAs targeting *lncR492*. Both esiRNAs achieved a knock-down efficiency of >60% (Fig 1C) and were able to consistently reproduce the screening phenotype (Fig 1D).

**Fig 2:**
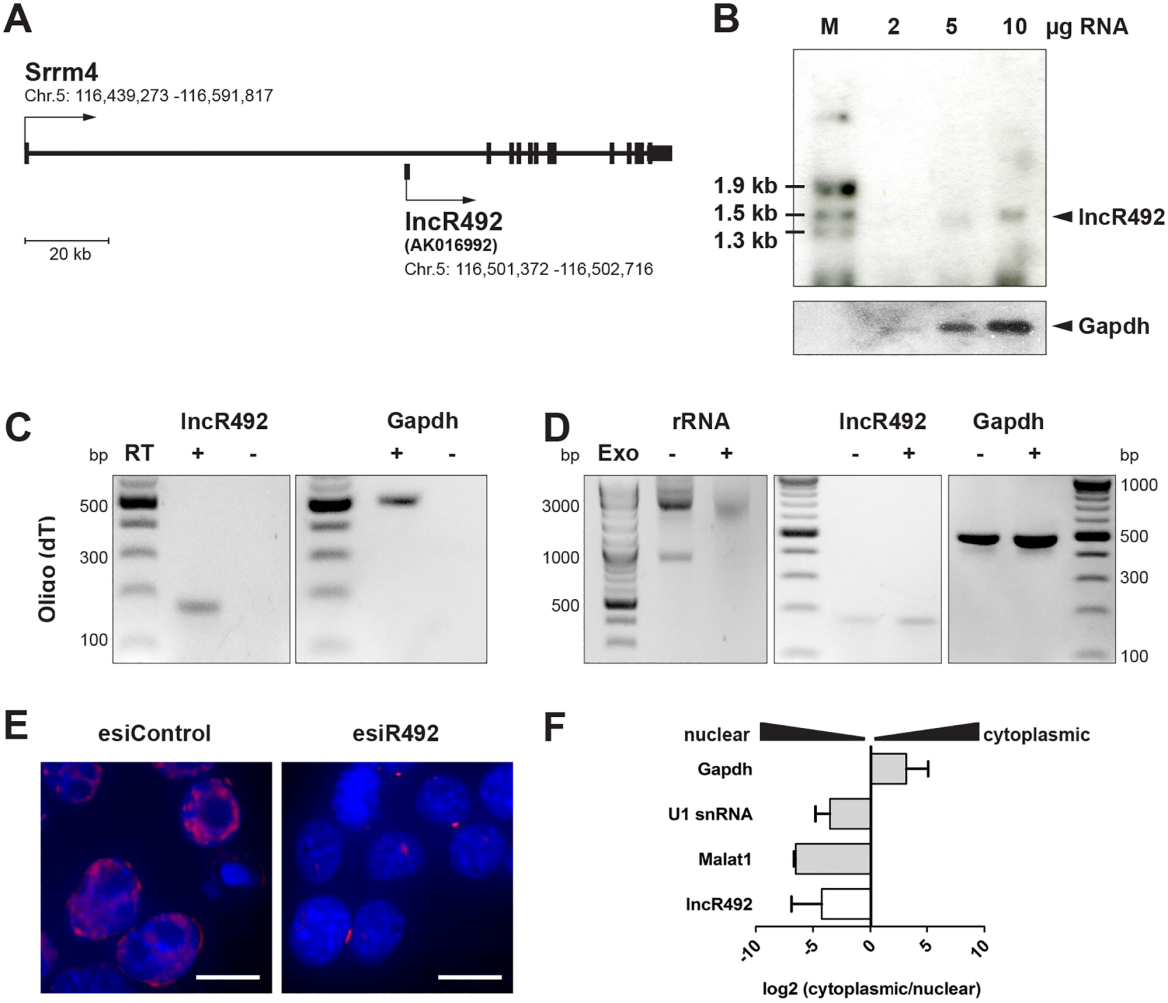
Characterization of *lncR492*. (A) Schematic of the lncR492 locus. *lncR492* (accession no. AK016992) is located within the first intron of the protein-coding gene *Srrm4*. (B) Northern blot of *lncR494*. Increasing amounts of total RNA were loaded. Black arrow indicates *lncR492*-specific signal at ~1400 bp. A probe targeting *Gapdh* mRNA was used as loading control. (C) Analysis of polyadenylation. mRNA was transcribed into cDNA by using Oligo(dT) primer followed by PCR. (D) RT-PCR analysis of *lncR492* and *Gapdh* expression after the RNA extract was treated with a 5’-phosphate-dependent exonuclease, which results in a degradation of f.ex. ribosomal RNA (left panel). (E) RNA FISH of *lncR492* expression in undifferentiated ESC. The *lncR492*-specific signal (red) was reduced after *lncR492* knock-down. Scale bars are 10 μm. (F) Cellular fractionation in ESC was followed by RNA isolation and mRNA expression analysis by qRT-PCR.

Most of the lncRNAs have been identified by computational annotation. Thus, they are classified as >200 nucleotides long transcripts with no coding potential. Coding potential calculators have been utilized to score the coding potential of *lncR492*. CPC [21] and CPAT [22] analysis resulted in a negative coding potential score (-1.17) and a low coding probability (0.05) for *lncR492*, respectively (S1 Fig), supporting the non-coding assignment. *LncR492* is located within the first intron of the protein-coding gene *Srrm4*, a splicing factor involved in the development of the nervous system (Fig 2A) [23, 24]. Due to its intronic location we first wanted to exclude that *lncR492* is an alternative exon of the host protein-coding gene. Northern blot analysis with a *lncR492*-specific probe identified a single band of approx. 1.4 kb (Fig 2B), confirming that *lncR492* is an *Srrm4*-independent transcript.

Like mRNAs many lncRNAs are poly(A)-tailed and capped [5, 6]. cDNA synthesis using Oligo(dT) primers confirmed that *lncR492* has a poly(A) tail (Fig 2C). Furthermore, we were able to amplify *lncR492* after exonuclease treatment, verifying the existence of RNA capping (Fig 2D). To further characterize and attempt a functional description, we investigated its cellular localization. Fluorescent in situ hybridization (FISH) showed a predominant nuclear localization of *lncR492*. The specificity of the FISH signal was confirmed by a loss of signal after *lncR492* knock-down (Fig 2E). Consistent with FISH, nuclear fractionation followed by qRT-PCR analysis confirmed an enrichment of *lncR492* in the nuclear lysate together with *U1 snRNA* and *Malat1* mRNA, whereas the coding mRNA of *Gapdh* was enriched in the cytoplasmic fraction (Fig 2F).

Thus, the esiRNA-based screen in differentiating ESCs identified *lncR492* as a non-coding nuclear transcript, which might play a role in regulating ectodermal cell fate commitment.

### *lncR492* impedes ESC differentiation in a lineage specific manner

**Fig 3:**
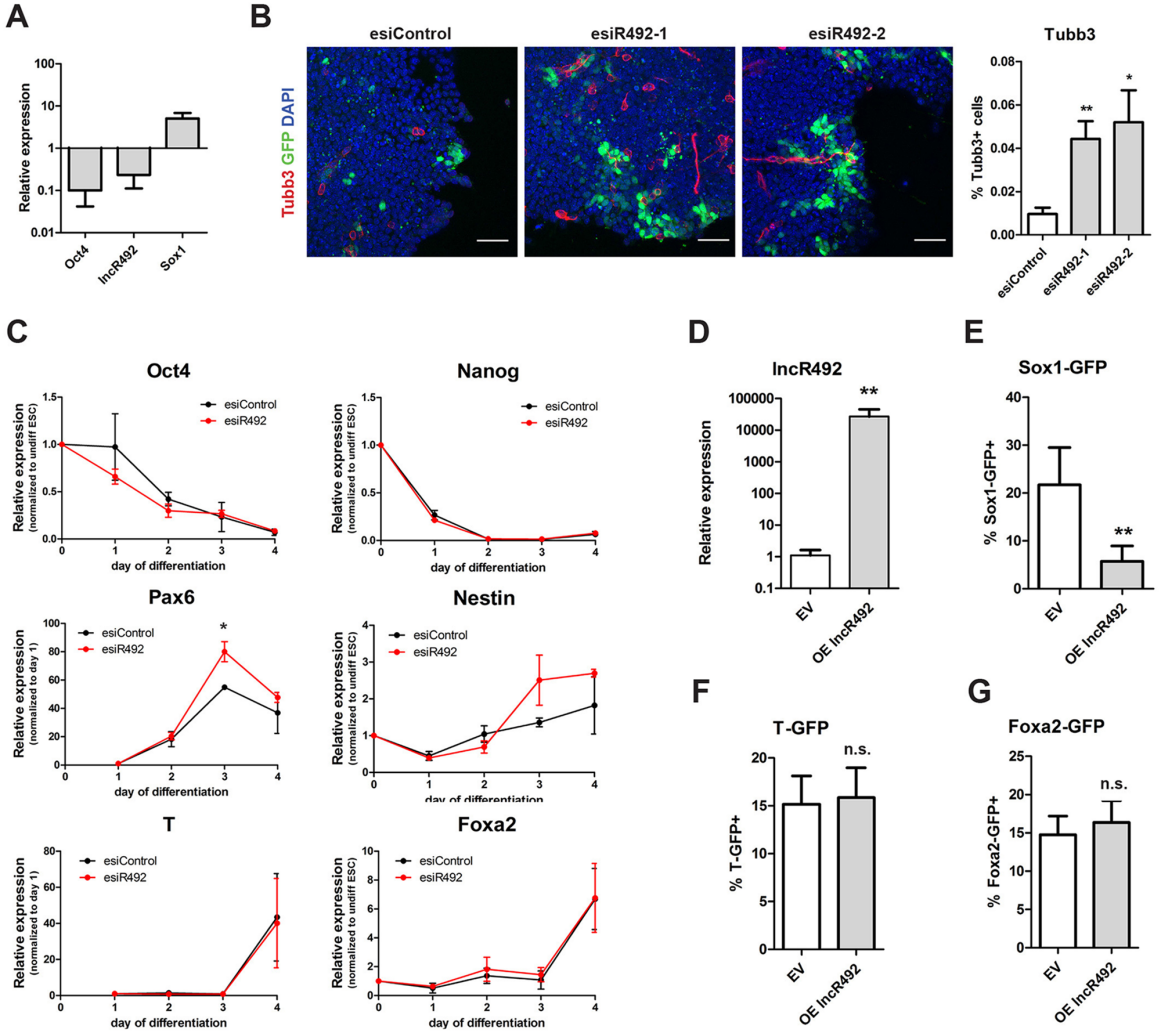
*LncR492* inhibits specifically neural differentiation. (A) Gene expression analysis by qRT-PCR in Sox1-GFP ESCs during 4 days of differentiation in N2B27. Data presents the mean ± SD of three independent experiments. (B) Immunofluorescent analysis of Sox1-GFP ESC after 72h of differentiation. Endogenous GFP (green), Tubb3 (red) and DAPI (blue) are shown. Scale bar 50 μm. Bar graph shows quantification of Tubb3 positive cells, which were normalized to the overall cell number. (C) Time course analysis of gene expression by qRT-PCR after *lncR492* knock-down in Sox1-GFP ESCs. Data presents mean ± SD of three independent experiments. (D) Transient overexpression of *lncR492* analysed by qRT-PCR in Sox1-GFP ESCs. Data presents the mean ± SD of three independent experiments. EV—empty vector. (E) FACS analysis of % Sox1-GFP positive cells after overexpression of *lncR492* and 4 days of differentiation. Data presents mean ± SD of 4 independent experiments. (F) FACS analysis of % T-GFP positive cells after overexpression of *lncR492* and 4 days of differentiation. Data presents mean ± SD of 4 independent experiments. (G) FACS analysis of % Foxa2-GFP positive cells after overexpression of *lncR492* and 4 days of differentiation. Data presents mean ± SD of 4 independent experiments. * p<0.05; ** p<0.01; *** p<0.001; n.s.–not significant.

The screening result suggested that *lncR492* acts as an inhibitor of ectodermal differentiation. To clarify its role and specificity in lineage commitment, we first tested its expression in pluripotent and differentiating ESCs. These results revealed that *lncR492* has its highest expression in the pluripotent state. Interestingly, together with *Oct4*, *lncR492* is down-regulated during neural differentiation (Fig 3A). To further investigate lineage specific effects of *lncR492* expression, we utilized fluorescently labeled lineage specific ESC reporter lines that allowed us to FACS-sort Sox1-GFP+ (ectoderm), Brachyury-GFP+ (T, mesoderm) and Foxa2-GFP+ (endoderm) cells after differentiation. In accordance with the reporter, the lineage specific genes were up-regulated when the cells were cultured under differentiation conditions in N2B27 alone (Sox1-GFP) or supplemented with 30 μg/ml Activin A (Foxa2-GFP) or 10 μg/ml BMP4 (T-GFP; S2A Fig). Interestingly, we saw that only in Sox1-GFP+ cells *lncR492* expression was reduced whereas in mesodermal and endodermal committed cells the expression was unaltered compared to the pluripotent state (S2A Fig), indicating that *lncR492* might have a distinct role in ESC differentiation towards the ectodermal lineage.

A closer look at the time course of differentiation towards ectoderm reveals that *lncR492* is already down-regulated after 24h and keeps its low expression the entire differentiation process. Importantly, *Srrm4* shows the same down-regulation as *lncR492* after 24 h but instead of maintaining the low expression, *Srrm4* is up-regulated continuously (S2B Fig). This result supports further the independence of *lncR492* from its host gene *Srrm4*.

The ectoderm-specific action of *lncR492* was further supported by an increased abundance of beta-3-tubulin (Tubb3)-positive cells in the *lncR492* knock-down condition compared to cells treated with a control esiRNA as seen by immunofluorescent staining (Fig 3B). Additionally, we analysed the gene expression of pluripotency as well as germ layer specific genes during the time course of differentiation after lncRNA knock-down. Knock-down of *lncR492* accelerated the loss of *Oct4* gene expression slightly without affecting *Nanog* (Fig 3C). In accordance with the initially observed inductive effect on Sox1, *lncR492* knock-down resulted also in an increased expression of the neural markers *Pax6*, and *Nestin* from day 3 onwards, whereas the mesendodermal lineage markers *T* and *Foxa2* were not affected (Fig 3C). Hence, our knock-down experiments suggest that loss of *lncR492* is required to enable differentiation towards the neural lineage.

Next we wanted to test whether overexpression of *lncR492* is sufficient to block neural commitment. To test this hypothesis, we transfected the Sox1-GFP ESCs with a plasmid that overexpressed *lncR492* (Fig 3D) and plated the cells under conditions that promote differentiation (Fig 1A). The percentage of Sox1-GFP+ cells dropped significantly from over 20% to roughly 5% unmasking the ability of *lncR492* to block neural differentiation (Fig 3E). In contrast, overexpression of *lncR492* had no effect on T-GFP and Foxa2-GFP differentiation (Fig 3F and 3G), demonstrating that overexpression of *lncR492* does not per se block ESC differentiation. To confirm that the observed phenotypes are not cell line specific, we repeated the knockdown and overexpression experiments in a second mouse ES cell line (R1/E) and obtained similar results (S2C and S2D Fig).

To verify that *lncR492* is not required for the maintenance of ESC stemness, we tested whether its mis-expression affects Oct4 expression under self-renewing conditions. To address this, Oct4-GFP ESCs were first cultured and transfected in N2B27 medium supplemented with 2i+LIF. 48h post transfection, neither *lncR492* knockdown, nor overexpression showed any changes in Oct4-GFP expression (S2E Fig). To exclude that the result was dependent on the chosen self-renewal culture condition, we repeated the experiment with Oct-GFP cells cultured in the presence of FCS+LIF. Again neither knock-down nor overexpression altered the number of Oct4-GFP positive cells. In contrast, knock-down of Rad21 reduced the number of Oct4-GFP positive cells, signifying loss of pluripotency (S2F Fig) [20]. Thus, we were able to confirm that *lncR492* is not involved in the maintenance of pluripotency but that it has its regulatory function in the lineage specific inhibition of ESC differentiation towards the neural lineage.

### *lncR492* interacts with HuR and inhibits neuroectodermal differentiation by activating Wnt signaling

Several lncRNAs have been shown to regulate their neighboring genes [25]. To test whether this is true for *lncR492*, we tested the effect of *lncR492* knock-down and overexpression on *Srrm4* expression. Experiments employing two independent esiRNAs targeting *lncR492* as well as overexpression of *lncR492* did not significantly change the mRNA levels of *Srrm4* (S3A Fig), indicating that *lncR492* exerts its function without regulating the expression of *Srrm4*. To search for other possible mechanisms of *lncR492* action we employed a proteomic approach.

**Fig 4:**
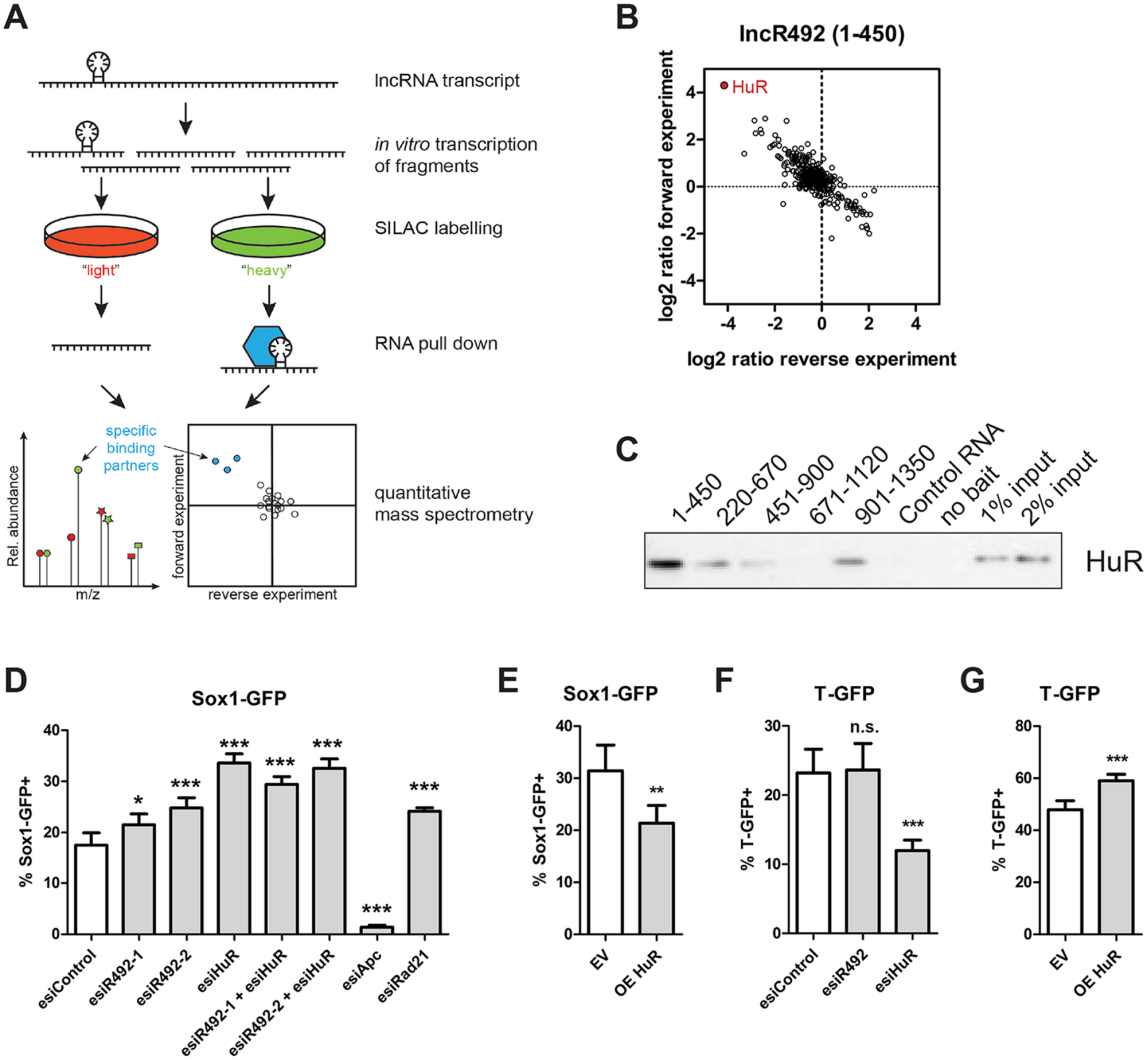
HuR regulates ectodermal differentiation similar to *lncR492*. (A) Schematic overview over mass spectrometry after RNA pull-down. (B) Two-dimensional interaction blot for 1-450bp lncR492 mRNA fragment incubated with ESC total cell lysate identifies HuR (Elavl1) as possible binding partner. (C) Western blot against HuR after RNA pull down. (D) FACS analysis of % Sox1-GFP positive cells after esiRNA transfection and 4 days of differentiation. Knock-down of *lncR492* and HuR alone or in combination. Apc and Rad21 were targeted as controls. Data presents mean ± SD of 4 independent experiments. (E) FACS analysis of % Sox1-GFP+ cells after overexpression of HuR. Data presents mean ± SD of 4 independent experiments. (F) FACS analysis of % T-GFP+ cells after knock-down of HuR. Data presents mean ± SD of 4 independent experiments. (G) FACS analysis of % T-GFP+ cells after overexpression of HuR. Data presents mean ± SD of 4 independent experiments. * p<0.05; ** p<0.01; *** p<0.001; n.s.–not significant.

To identify putative proteins that physically interact with *lncR492*, we used SILAC-based RNA pull-downs followed by mass spectrometry to identify proteins bound to the *lncR492* transcript (Fig 4A). We generated five 450 bp long overlapping fragments of *lncR492* by *in vitro* transcription. As negative control for the RNA pull-downs a fragment of the well characterized *PTPN13* mRNA with exactly the same size was chosen [26]. For each of the five overlapping fragments proteins were enriched as putative specific interaction partners (S3B Fig, S2 Table). Among the strongest *lncR492*-binding candidates was HuR (encoded by *Elavl1*), which was most enriched in pull downs with fragment 1 (1–450 bp, Fig 4B) as well as fragment 4 (671–1130 bp, S3B Fig, S2 Table). Western blot analysis indeed confirmed the interaction of HuR with fragment 1 (Fig 4C). Repeating this western blot produced virtually identical results (data not shown). HuR is a known mRNA binding protein involved in both, mRNA decay and stabilization, [27, 28] and has been described in supporting lncRNA function during muscle differentiation [29] and in cancerogenesis [28]. Thus, we decided to test whether HuR is involved in *lncR492*’s role as ectodermal inhibitor.

HuR has been shown to bind to ARE-containing (adenylate–uridylate-rich elements) mRNAs [30] and RNA-protein interaction studies have identified additionally uracil-rich motifs specific for HuR mRNA binding [31, 32]. Our sequence analysis of *lncR492* revealed two distinct uracil-rich regions, where we were able to map several of the described heptameric or 17- to 20-base long motifs. In the 5’ region a 57 bp long motif-containing sequence (HuR BS1) was identified as well as an 11 bp long sequence in the 3’ region (HuR BS2) of the transcript (S3C Fig). The position of HuR BS1 correlates with the HuR enrichment in the RNA pull-down experiment, as it is located within the first fragment (1–450 bp). Assuming that *lncR492* and HuR act in a complex, we wanted to test whether knock-down of HuR results in a similar phenotype as the *lncR492* knock-down. EsiRNA mediated knock-down as well as overexpression of HuR was confirmed by western blot (S3D Fig). ESC differentiation in N2B27 after HuR knock-down resulted in an increase of Sox1-GFP+ cells, similar to what was observed after *lncR492* knock-down (Fig 4D). Importantly, *lncR492* overexpression was able to reverse the knock-down phenotype in Sox1-GFP ESC differentiation (Fig 3E). Likewise overexpression of HuR was capable of reducing the differentiation into Sox1-GFP+ cells significantly (Fig 4E). In accordance with the *lncR492* results, differentiation into Foxa2-GFP+ cells was not affected by HuR mis-expression (S3E Fig). However, knock-down and overexpression of HuR reduced and increased T-GFP expression, respectively (Fig 4F and 4G). Given that *lncR492* knock-down and overexpression did not affect differentiation into T-GFP+ cells, this result suggests additional functions of HuR with an *lncR492*-independent mechanism here. Similar to *lncR492*, HuR is not required to maintain self-renewal, as the pluripotency marker Oct4 was not altered after HuR knock-down or overexpression (S3F Fig). Hence, phenotypic consequences after HuR knockdown and overexpression on the Sox1-GFP reporter mirrored the *lncR492* phenotype to a large extend, suggesting that *lncR492* and HuR are interlinked.

**Fig 5:**
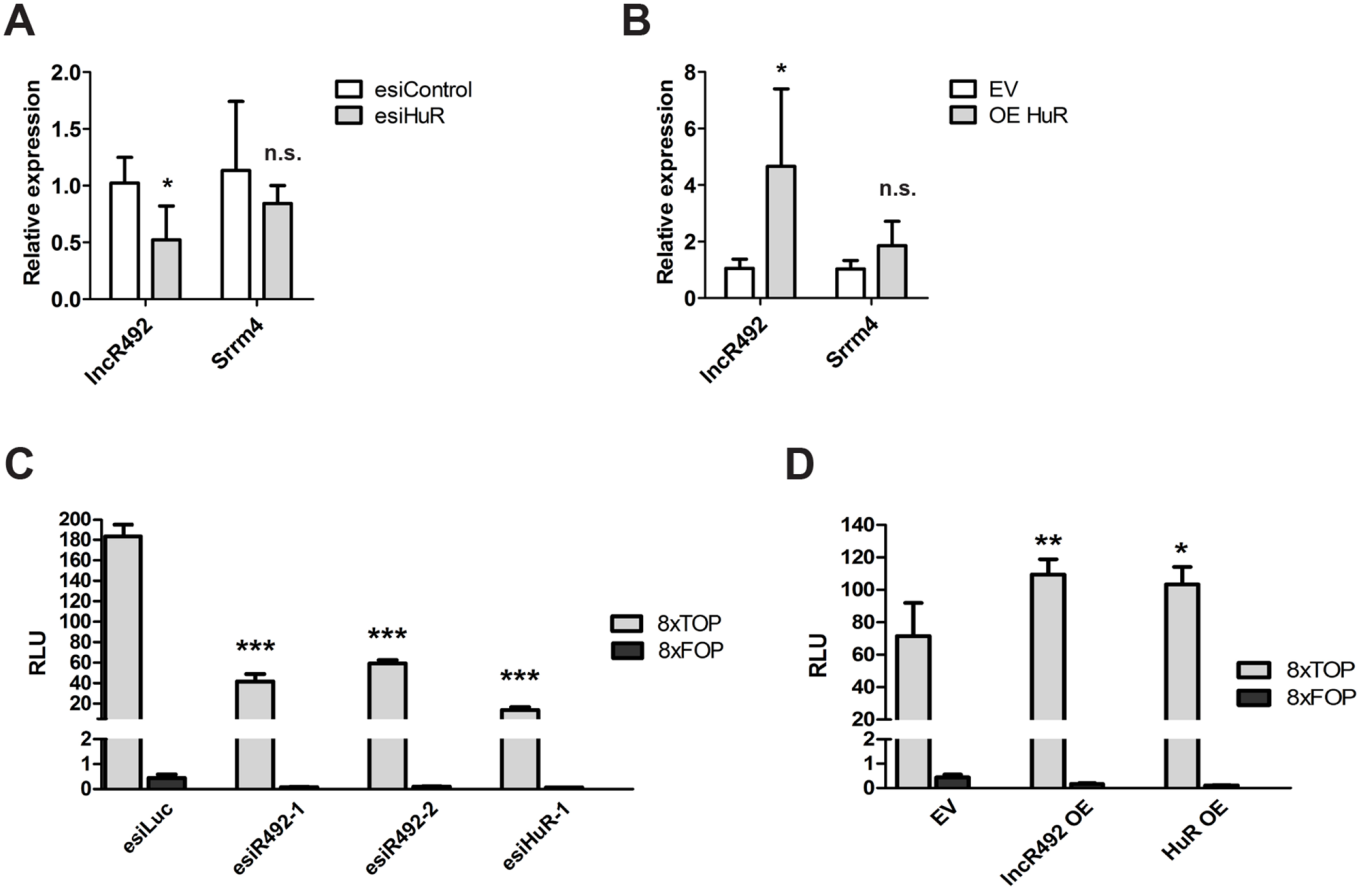
*LncR492* cooperates with HuR by activation of Wnt signaling. (A) QRT-PCR analysis of *lncR492* and *Srrm4* after HuR knock-down. Data presents the mean ± SD of four independent experiments. (B) QRT-PCR analysis of *lncR492* and *Srrm4* after HuR overexpression. Data presents the mean ± SD of four independent experiments. EV—empty vector. (C) Luciferase assay for Wnt signaling using the TOP-Flash and FOP-Flash luciferase construct after esiRNA transfection. Data presents mean ± SD of three independent experiments. (D) Luciferase assay for Wnt signaling after *lncR492* or HuR overexpression. Data presents mean ± SD of three independent experiments. * p<0.05; ** p<0.01; *** p<0.001; n.s.–not significant.

Due to HuR’s potential to bind mRNA as well as non-coding RNA, we next wanted to verify whether HuR has an impact on *lncR492* gene expression. Interestingly, knock-down and overexpression of HuR resulted in a respective decrease and increase of *lncR492*, whereas *Srrm4* transcript levels were not affected (Fig 5A and 5B). These results suggest that binding of HuR to *lncR492* regulates the stability of *lncR492*.

Among its broad spectra of action, HuR has also been shown to regulate Wnt signaling in a context dependent manner. HuR was shown to block Wnt5a signaling in human breast cancer by direct inhibition of translation [33], whereas in the intestinal epithelium HuR supports Wnt signaling by stabilizing LRP6 mRNA [34]. Together with the known inhibitory effect of Wnt signaling on neural differentiation, we finally tested, whether *lncR492* together with HuR have an impact on this signaling pathway. We utilized the TOPFlash luciferase reporter assay to monitor canonical Wnt signaling after *lncR492* and HuR mis-expression. Both, knock-down of *lncR492* as well as HuR reduced Wnt signaling significantly (Fig 5C). In contrast, overexpression of *lncR492* and HuR enhanced Wnt signaling (Fig 5D), confirming, that *lncR492* together with HuR augments Wnt signaling, which in return might exert the inhibitory effect on neural differentiation in ESCs.

## Discussion

RNAi based libraries targeting protein coding as well as non-coding genes have been valuable tools to study gene function in pluripotent ESC [8, 9, 14, 35]. Our present work establishes that loss-of-function screens for lncRNAs via RNAi are also feasible in differentiating ESCs. Taking advantage of the Sox1-GFP reporter cell line, we were able to identify lineage specific lncRNAs regulating ectodermal differentiation. Notably, our hits did not score in a pluripotency screen with the same library in ESC [9] demonstrating further applications for RNAi-based lncRNA screens in the lineage transition, here towards the ectoderm. Reporter cell lines for the endodermal and mesodermal lineages could be used in the future to complement the dataset for the first lineage commitment in ESC relating to gastrulation. This might not only allow for the identification of lineage specific lncRNAs, but also help to discover lncRNAs that regulate differentiation more globally.

Many functionally studied lncRNAs have been shown to regulate their neighboring genes [7, 36]. *LncR492* is located within the first intron of the protein-coding gene *Srrm4*. However, knock-down and overexpression of the lncRNA did not affect *Srrm4* expression. In fact, *Srrm4* is hardly expressed in ESC and was only marginal up-regulated after 4 days of differentiation (S2B Fig), which is in accordance with literature, where *Srrm4* has been shown to play a role later on during neurogenesis [23]. Interestingly, Srrm4 was assigned the function of regulating alternative splicing [23, 24], a cellular function that has been shown to involve lncRNAs such as *Malat1* and *Pnky* [37, 38]. Therefore, it might be interesting to investigate whether there exists a functional relation between *lncR492* and *Srrm4* in later developmental stages and whether this includes alternative splicing.

In our study, we identified and validated the mRNA binding protein HuR as interaction partner of *lncR492* during the regulation of neural differentiation. Yet, *lncR492* is not the first lncRNA that has been described to act in concert with HuR [27–29, 39–42]. All these studies including ours demonstrate that lncRNAs are essential in specifying the large repertoire of functions that HuR can fulfill. Thereby, HuR can have a stabilizing or destabilizing property depending on the spatial and temporal context. This highlights once more the regulatory function of lncRNAs. Interestingly, several other proteins known to be involved in regulating ESC identity and differentiation were also identified to interact with *lncR492* in our mass spec analysis, suggesting that protein interactions of this lncRNA could be complex. Additional work will be required to unravel the protein interaction network of *lncR492*.

Finally, we could show that both, *lncR492* and HuR, are involved in maintaining Wnt signaling, as knock-down and overexpression of both genes resulted in a decrease and increase of Wnt signaling, respectively. *In vitro* studies in ESC have shown that the upregulation of Wnt antagonist like Dkk1 and Sfrp2 are required for neuroectodermal differentiation [17, 18]. Also members of the Tcf/Lef protein family were shown to balance self-renewal and differentiation as down-stream effectors of Wnt signaling [43–45]. Further studies are required to elucidate the molecular mechanism by which *lncR492* in concert with HuR control Wnt signaling during the transition from pluripotency to neuroectodermal differentiation.

## Supporting information

S1 Fig The coding potential of *lncR492* was tested by utilizing the coding potential calculator (CPC; Kong et al., 2007) and the coding potential assessment tool (CPAT; Wang et al., 2013). (TIF)

S2 Fig (A) *LncR492* expression in differentiated ESCs. Therefore T-GFP and Foxa2-GFP reporter cells were differentiated N2B27-containing medium supplemented with 10 μg/ml BMP4 or 30μg/ml ActivinA for 4 days, respectively. Sox1-GFP ESCs were differentiated for 4 days in medium containing the serum replacement N2B27 only. GFP+ cells were sorted by FACS, RNA was isolated and *lncR492* as well as lineage specific gene expression was analysed by qRT-PCR. Expression was normalized to undifferentiated ESC. Data presents mean ± SD of three independent experiments.
(B) QRT-PCR analysis of *lncR492* and *Srrm4* during the time course of differentiation. Data presents mean ± SD of three independent experiments.
(C) Western blot analysis of Tubb3 after *lncR492* knock-down or overexpression in T-GFP ESCs. Bar graph represents the quantification of three independent western blot experiments. Data presents the mean ± SD.
(D) Gene expression analysis of the T-GFP reporter ESC (R1/E) by qRT-PCR after *lncR492* knock-down or overexpression. Cells were harvested after 4 days of differentiation in N2B27. Data presents the mean ± SD of three independent experiments.
(E) FACS analysis of GFP expression after *lncR492* knock-down and overexpression in Oct4-GFP ESC cultured in N2B27+2i+LIF medium. Data represents mean ± SD of four independent experiments.
(F) FACS analysis of GFP expression after *lncR492* knock-down and overexpression in Oct4-GFP ESC cultured in medium supplemented with FCS+LIF. Knock-down of Rad21 was used as a positive control. Data represents mean ± SD of four independent experiments.
* p<0.05; ** p<0.01; *** p<0.001; n.s.–not significant.
(TIF)

S3 Fig (A) *Srrm4* expression after *lncR492* knock-down and overexpression measured by qRT-PCR. Data represents mean ± SD of three independent experiments.
(B) Summary table of proteins detected by mass spectrometry analysis. The lncRNA transcript was split into five overlapping fragments of 450 bp length each. The top ten putative interaction proteins for each lncRNA fragment are listed according to their abundance.
(C) Nucleic acid sequence (mRNA) of *lncR492*. Putative binding sides for HuR are highlighted in red based on the consensus sequence NNUUNNUUU.
(D) Western blot of HuR knock-down and overexpression. Gapdh was used as loading control. EV—empty vector.
(E) FACS analysis of Foxa2-GFP expression after *lncR492* and HuR knock-down or HuR overexpression. Cells were differentiated for 4 days in N2B27 supplemented with 30 ng/ml ActivinA. Data presents mean ± SD of three independent experiments.
(F) FACS analysis of Oct4-GFP expression 48h after HuR knock-down and overexpression. Oct4-GFP cells were cultured in N2B27+2i+LIF medium. Data presents mean ± SD of three independent experiments.
* p<0.05; ** p<0.01; *** p<0.001; n.s.–not significant.
(TIF)

S1 Table Summary of the screen results. Z-scores of the primary and the validation screen are shown for each replicate. Hits of the primary screen with an average Z-score >3 are highlighted in green (increasing the number of Sox1-GFP positive cells) and hits with an average Z-score < -3 are highlighted in orange (decreasing the number of Sox1-GFP positive cells). In the validation screen a Z-score > 2 or <-2 are considered as hit and highlighted in green.
(XLSX)

S2 Table Summary table of the mass spectrometry after *lncR492* pull down. Identified proteins for each fragment used in the pull-down experiment are shown.
(XLSX)
