# Supplementary figures and images for: The long noncoding RNA lncR492 inhibits neural differentiation of murine embryonic stem cells
Source: PLoS One. 2018 Jan 24;13(1):e0191682. doi: 10.1371/journal.pone.0191682 (PMC5783419; doi:10.1371/journal.pone.0191682)

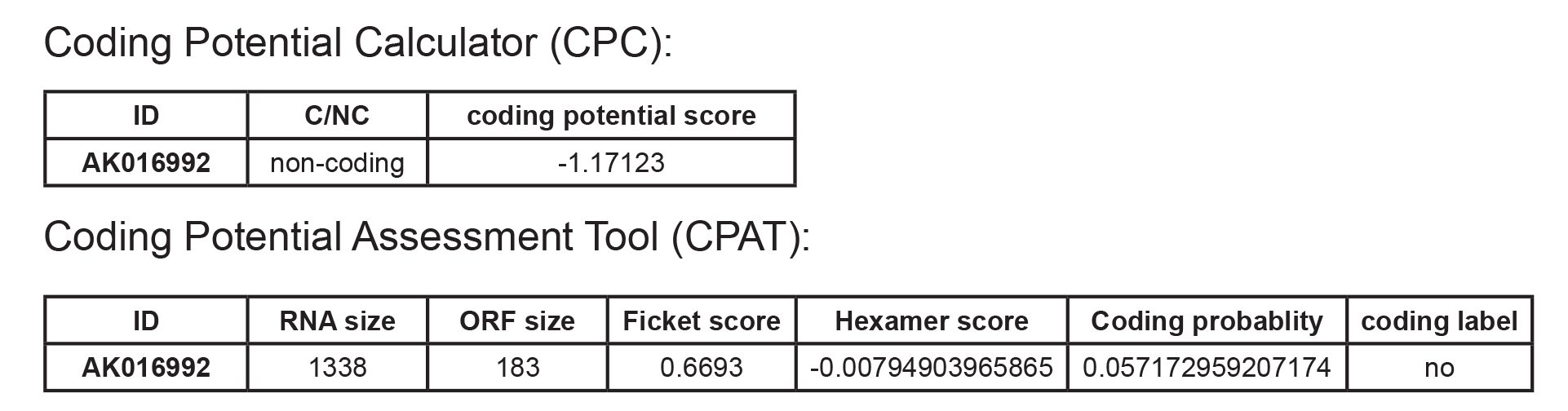

Supplement: S1 Fig — (TIF) [file pone.0191682.s001.tif]

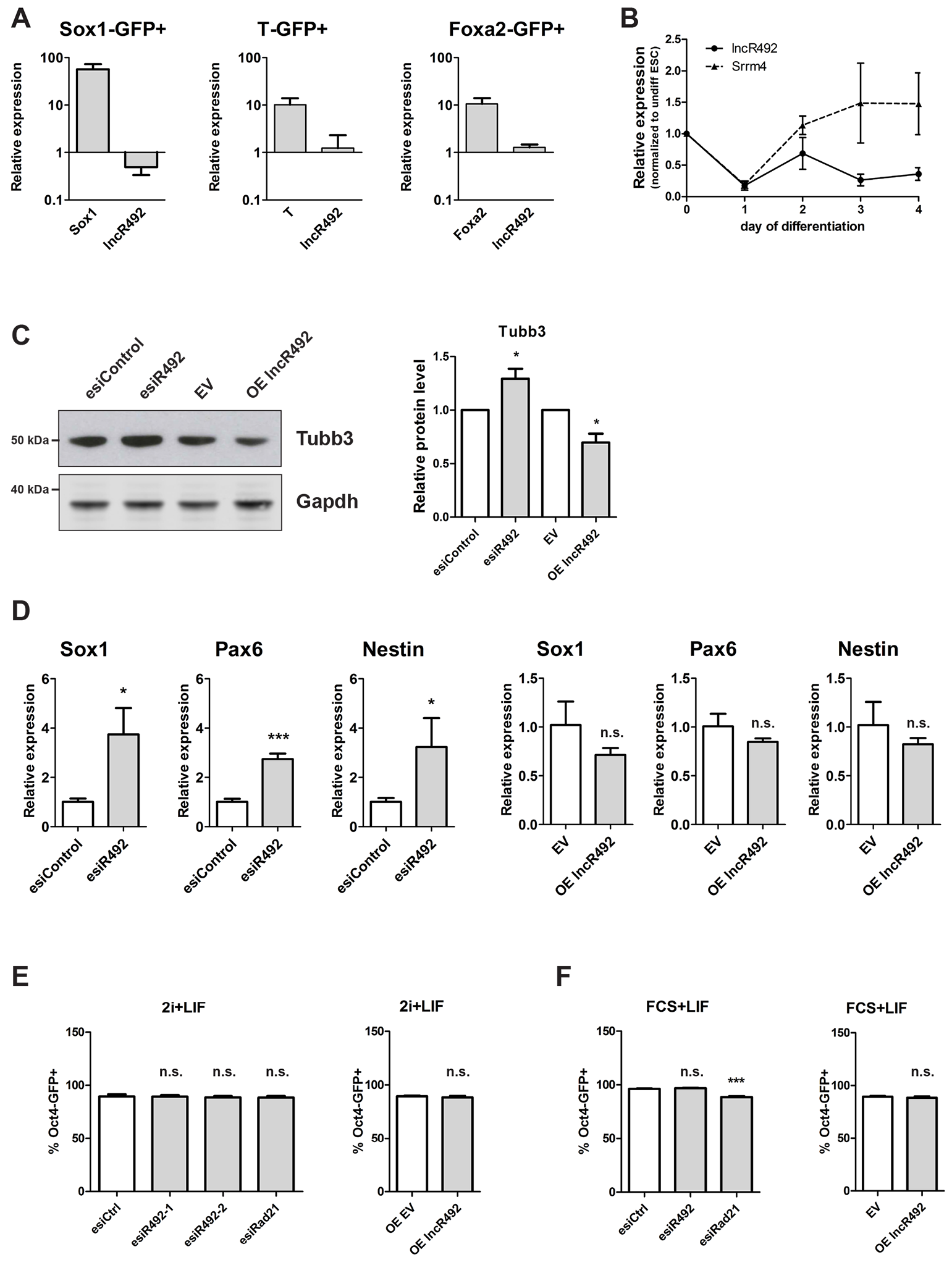

Supplement: S2 Fig — (A) LncR492 expression in differentiated ESCs. Therefore T-GFP and Foxa2-GFP reporter cells were differentiated N2B27-containing medium supplemented with 10 μg/ml BMP4 or 30μg/ml ActivinA for 4 days, respectively. Sox1-GFP ESCs were differentiated for 4 days in medium containing the serum replacement N2B27 only. GFP+ cells were sorted by FACS, RNA was isolated and lncR492 as well as lineage specific gene expression was analysed by qRT-PCR. Expression was normalized to undifferentiated ESC. Data presents mean ± SD of three independent experiments. (B) QRT-PCR analysis of lncR492 and Srrm4 during the time course of differentiation. Data presents mean ± SD of three independent experiments. (C) Western blot analysis of Tubb3 after lncR492 knock-down or overexpression in T-GFP ESCs. Bar graph represents the quantification of three independent western blot experiments. Data presents the mean ± SD. (D) Gene expression analysis of the T-GFP reporter ESC (R1/E) by qRT-PCR after lncR492 knock-down or overexpression. Cells were harvested after 4 days of differentiation in N2B27. Data presents the mean ± SD of three independent experiments. (E) FACS analysis of GFP expression after lncR492 knock-down and overexpression in Oct4-GFP ESC cultured in N2B27+2i+LIF medium. Data represents mean ± SD of four independent experiments. (F) FACS analysis of GFP expression after lncR492 knock-down and overexpression in Oct4-GFP ESC cultured in medium supplemented with FCS+LIF. Knock-down of Rad21 was used as a positive control. Data represents mean ± SD of four independent experiments. * p<0.05; ** p<0.01; *** p<0.001; n.s.–not significant. (TIF) [file pone.0191682.s002.tif]

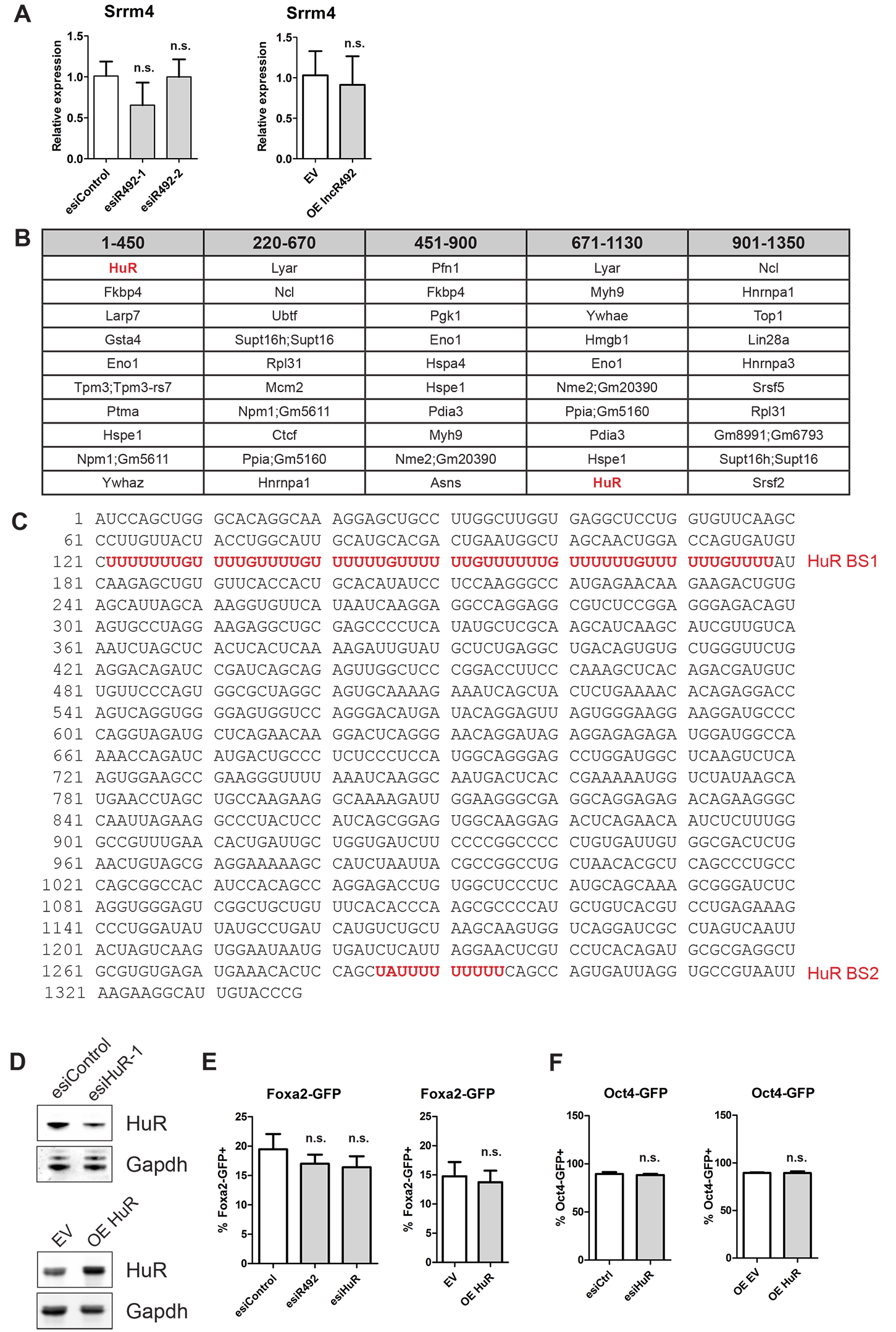

Supplement: S3 Fig — (A) Srrm4 expression after lncR492 knock-down and overexpression measured by qRT-PCR. Data represents mean ± SD of three independent experiments. (B) Summary table of proteins detected by mass spectrometry analysis. The lncRNA transcript was split into five overlapping fragments of 450 bp length each. The top ten putative interaction proteins for each lncRNA fragment are listed according to their abundance. (C) Nucleic acid sequence (mRNA) of lncR492. Putative binding sides for HuR are highlighted in red based on the consensus sequence NNUUNNUUU. (D) Western blot of HuR knock-down and overexpression. Gapdh was used as loading control. EV—empty vector. (E) FACS analysis of Foxa2-GFP expression after lncR492 and HuR knock-down or HuR overexpression. Cells were differentiated for 4 days in N2B27 supplemented with 30 ng/ml ActivinA. Data presents mean ± SD of three independent experiments. (F) FACS analysis of Oct4-GFP expression 48h after HuR knock-down and overexpression. Oct4-GFP cells were cultured in N2B27+2i+LIF medium. Data presents mean ± SD of three independent experiments. * p<0.05; ** p<0.01; *** p<0.001; n.s.–not significant. (TIF) [file pone.0191682.s003.tif]
